# Novel Wearable-Based Real-Time Temperature Monitoring in Hospitals for Febrile Adverse Events in Patients with Cancer: A Prospective Feasibility Study

**DOI:** 10.3390/s25237111

**Published:** 2025-11-21

**Authors:** Yun Kwan Kim, Seo-Yeon Ahn, Sun Jung Lee, Chae-Bin Song, YeWon Hong, Ray Kim, Hee Jo Baek, Hoon Kook, Mihee Kim, Ga-Young Song, Ho Cheol Jang, Sung-Hoon Jung, Deok-Hwan Yang, Je-Jung Lee, Hyeoung-Joon Kim, Gyung Chul Kim, Hoo Hyun Kim, Young-Shin Lee, Jae-Sook Ahn

**Affiliations:** 1 Department of Research and Development, SEERS Technology Co., Ltd., Pyeongtaek 17707, Gyeonggi-do, Republic of Korea; ykwin@korea.ac.kr (Y.K.K.); michelle.lee@seerstech.com (S.J.L.); claire.song@seerstech.com (C.-B.S.); ray.kim@seerstech.com (R.K.); eric.kim@seerstech.com (G.C.K.); kei.kim@seerstech.com (H.H.K.); 2 Department of Hematology-Oncology, Chonnam National University Hwasun Hospital, Hwasun-gun 58128, Jeollanam-do, Republic of Korea; armful12@hanmail.net (S.-Y.A.); mihi4300@hanmail.net (M.K.); songga0e@naver.com (G.-Y.S.); minjokobject@gmail.com (H.C.J.); shglory@hanmail.net (S.-H.J.); drydh1685@hotmail.com (D.-H.Y.); drjejung@chonnam.ac.kr (J.-J.L.); hjoonk@chonnam.ac.kr (H.-J.K.); 3 Strategic Planning Team, SEERS Technology Co., Ltd., Pyeongtaek 17707, Gyeonggi-do, Republic of Korea; sophia.hong@seerstech.com (Y.H.); luke.lee@seerstech.com (Y.-S.L.); 4 Department of Pediatrics, Chonnam National University Hwasun Hospital, Hwasun-gun 58128, Jeollanam-do, Republic of Korea; swan93@naver.com (H.J.B.); hoonkook@chonnam.ac.kr (H.K.)

**Keywords:** febrile neutropenia, real-time temperature monitoring, wearable device, cancer

## Abstract

Cancer remains a leading cause of mortality worldwide. Chemotherapy is effective but can cause febrile neutropenia, deplete neutrophils, and increase infection risk. Although timely fever detection is critical to prevent complications, periodic temperature monitoring has inherent limitations. Wearable devices (WDs) for real-time body temperature (RBT) monitoring are emerging as tools for early fever detection, though their clinical feasibility and usability remain unverified. This study assessed the clinical feasibility of a novel WD by comparing its temperature concordance and fever detection capability against a standard reference in patients with cancer. The RBT system’s clinical maturity, including its capacity for early fever detection and monitoring treatment-related changes, was evaluated. Furthermore, satisfaction among clinicians and patients was analyzed in a hospital setting. In this prospective study, patients with hematologic malignancies at high infection risk admitted to the Chonnam National University in Hwasun Hospital, South Korea, were enrolled. Data from 47 patients were analyzed to compare MT100D as WD (SEERS Technology Co., Ltd., South Korea) readings with axillary thermometers. Temperature concordance was evaluated using the intraclass correlation coefficient (ICC), and diagnostic performance was compared with the reference thermometer. The early fever detection performance of the MT100D was analyzed to evaluate the clinical effectiveness of the early response system. Usability was evaluated through a five-point Likert scale survey completed by clinical nurses. The MT100D demonstrated good agreement with the reference thermometer (mean difference: −0.19 ± 0.35 °C; ICC: 0.78). The RBT system achieved sensitivity, specificity, and area under the receiver operating characteristic curve values of 81.50%, 96.32%, and 96.60%, respectively. The early fever detection rate was 77.1%, with the RBT system detecting fever an average of 1.13 ± 1.28 h earlier than the reference. Usability and satisfaction assessments showed high patient satisfaction (mean 4.69 ± 0.5; range 4.57–4.80) and moderate clinician satisfaction (mean 3.63 ± 0.75; range 2.89–4.11). Among patients with cancer, including those at risk of febrile neutropenia, the MT100D demonstrated strong concordance with standard thermometry, enabling early fever detection. The RBT system shows promise for the early identification of neutropenic risk during chemotherapy.

## 1. Introduction

Cancer is one of the leading causes of mortality worldwide [1]. Recent advances in cancer biology and therapeutics have significantly expanded treatment options for solid tumors and hematologic malignancies [2]. However, increased use of therapies such as chemotherapy and chimeric antigen receptor T-cell (CAR-T) therapy has been accompanied by a rise in treatment-related complications, particularly febrile neutropenia (FN).

FN is a potentially life-threatening condition in which fever is a critical early indicator. FN affects thousands of patients annually and is associated with substantial healthcare costs and elevated infection-related mortality [3,4,5,6]. Timely fever detection is critical for prompt interventions, especially antibiotics, as delays are associated with worsened outcomes. Conventional temperature monitoring, typically every 4–8 h, may fail to detect early febrile episodes [7,8,9]. Despite reducing FN-related mortality, this approach remains suboptimal; delayed FN detection still results in bloodstream infection rates as high as 11–24% [10,11].

Recently, wearable devices (WDs) capable of continuous real-time body temperature (RBT) monitoring emerged as promising tools for early fever detection in hospitalized patients [12,13]. These systems can improve clinical responsiveness and outcomes through timely intervention. Prior studies have investigated WD-based RBT monitoring in selected populations: Verma et al. in autologous stem cell transplantation (ASCT) recipients [14], Flora et al. in hematopoietic stem cell transplant (HCT) and CAR-T therapy recipients [15], and case reports in pediatric patients with cancer and neutropenia [16].

However, most existing research has focused on homogeneous populations, such as ASCT or HCT recipients, or small groups of pediatric patients with cancer [14,15,16]. The performance and accuracy of WD-based RBT monitoring systems in more diverse, high-risk populations, including children and those receiving blood transfusions, remain underexplored. Furthermore, most trials have been limited to short-term monitoring (typically up to seven days), although many patients undergoing cancer treatment require prolonged hospitalization [16,17]. Therefore, the utility of RBT systems for extended fever surveillance and post-treatment monitoring is yet to be fully validated.

Although body temperature fluctuates with circadian rhythms and environmental influences, accurate and continuous monitoring is essential to detect clinically significant febrile events, particularly in vulnerable populations. This study compares the accuracy of absolute body temperature from intermittent and continuous temperature measurements and verifies the clinical effectiveness of continuous temperature monitoring in detecting temperature trends.

Therefore, the primary objectives of this study were (i) to compare the effectiveness of WD-based continuous temperature measurements versus intermittent body temperature measurements for early fever detection and agreement validation in patients with hematologic malignancies undergoing chemotherapy; (ii) to assess the clinical performance of an WD-based RBT monitoring system in terms of fever detection accuracy, early detection rate, and monitoring across treatment phases; and (iii) to assess user and clinician satisfaction, and ease of use of the WD and RBT monitoring system in a clinical setting.

## 2. Materials and Methods

### 2.1. Patients and Study Design

This prospective feasibility study was conducted between December 2023 and April 2024 at Chonnam National University Hwasun Hospital, Hwasun, South Korea. The study enrolled hospitalized patients weighing over 10 kg who required continuous temperature monitoring, including those with hematologic diseases or solid tumors requiring close fever surveillance.

**Figure 1:**
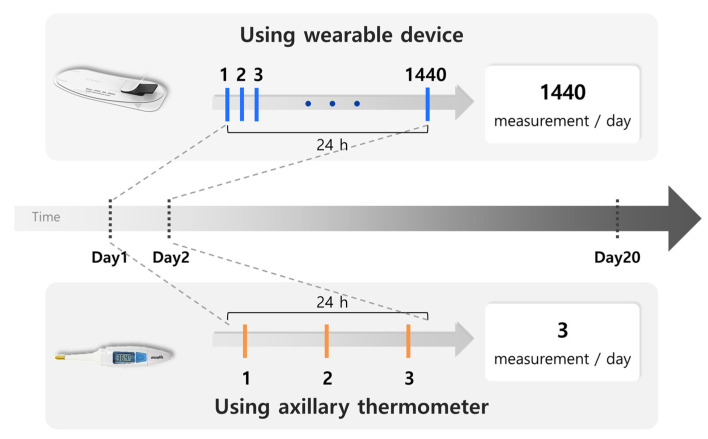
A schematic framework of the study timeline comparing reference and MT100D temperature measurements.

As illustrated in Figure 1, continuous temperature data recorded during febrile episodes were compared with corresponding values documented in hospital records using a consecutive sampling approach. Eligible participants were identified at admission to the hematology ward based on clinical evaluation. Final eligibility was confirmed by the attending hemato-oncologist based on the diagnosis and planned treatment regimen. Patients were considered at high risk for FN if they were scheduled to receive intensive chemotherapy expected to induce Grade ≥3 neutropenia or had preexisting neutropenia due to conditions such as severe aplastic anemia. Patients with solid tumors receiving myelosuppressive therapy were also included. Informed consent was obtained before enrollment. A trained research nurse provided instructions on device use and applied the MT100D patch (SEERS Technology Co., Ltd., Gyeonggi-do, South Korea) on the first monitoring day. Participants were selected based on pre-defined inclusion and exclusion criteria. Once enrolled, the MT100D patch was affixed, and continuous temperature monitoring commenced. The MT100D recorded the body temperature for each participant every minute, yielding 1440 measurements daily. Conversely, clinicians took axillary temperature measurements every eight hours. However, if temperature of patients reached 37.8 °C or higher as indicating fever, hourly follow-up measurements were performed by clinicians. The monitoring duration varied per patient, averaging 20 days (2–50 days) depending on individual clinical course and hospitalization length.

### 2.2. Sample Size

The sample size was calculated to evaluate the agreement between the widely used licensed axillary thermometer and the newly released MT100D. Estimation was performed using the G Power software, version 3.1 (Heinrich-Heine-Universität Düsseldorf, Düsseldorf, Germany) [17]. An effect size (Cohen’s d) of 0.5, reflecting a moderate difference (approximately 0.3–0.5 °C), was selected based on previous studies comparing wearable and standard thermometers in those febrile [18]. With a two-sided significance level (α) of 0.05 and a power (1-β) of 95%, the required sample size was calculated to be 42. Given a 20% attrition rate, the target enrollment was set at 50 participants.

### 2.3. Data Preparation

**Figure 2:**
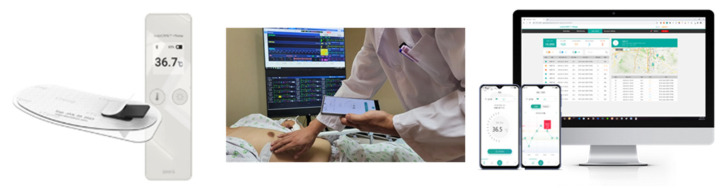
An illustration of the MT100D device and thynC^TM^ for real-time body temperature (RBT) monitoring. The left-hand image shows the MT100D device suitable for daily use; the center panel demonstrates RBT monitoring in a clinical environment, and the right panel displays the thynC^TM^ user interface.

Inclusion was based on the expected efficacy of combined treatments, guided by the Common Terminology Criteria for Adverse Events (CTCAE) Grade 4 for individual therapies [19]. Exclusion criteria included patients with fewer than 10 clinical records, body temperatures below 35.5 °C, and discharge within 15 min of modified vital sign measurement. Patients with conditions that could interfere with continuous patch adherence, such as adhesive allergies, atopic dermatitis, urticaria, or excessive sweating were also excluded. Investigators made additional exclusions for patients deemed clinically unsuitable. Detailed patient inclusion and exclusion results are presented in the Section 3 and illustrated in Figure 2.

The MT100D is factory-calibrated and provides an accuracy of ±0.1 °C within the clinical range (36.0–39.0 °C). It transmits temperature readings every 60 s to a central monitoring server using a dual-gateway wireless architecture via the 2.4 GHz ISM band. This configuration ensures uninterrupted signal transmission by maintaining simultaneous communication with two nearby gateways, even as patients move within the hospital. The device is rated IP57 for water resistance, supports extended battery life, and is designed for comfortable, prolonged use in pediatric and adult patients during routine activities.

### 2.4. RBT Monitoring System

**Figure 3:**
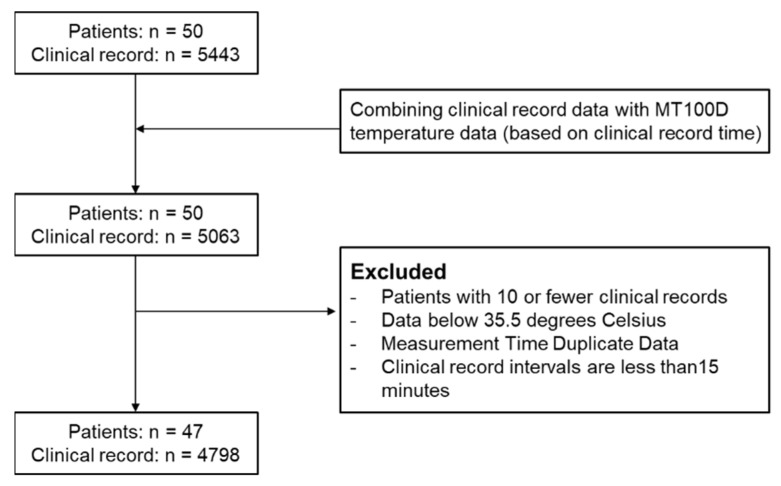
A patient inclusion and exclusion flow diagram for the prospective study.

The MT100D continuously records body temperature and transmits data in real time to a central monitoring platform (thynC™ system, SEERS Technology Co., Ltd., Pyeongtaek, Republic of Korea) in the oncology unit via a secure wireless network. This real-time monitoring system employs a dual-gateway wireless architecture, ensuring uninterrupted data transmission even as patients move within the oncology unit. In the single-connection setup, the temperature patch communicates with only one nearby gateway, which may result in transient signal loss when a patient moves out of range. However, the dual-connection approach allows the patch to communicate simultaneously with two nearby gateways. This configuration ensures that even if one connection is disrupted, the other remains active, thereby minimizing signal dropout and enhancing the reliability of continuous wireless temperature monitoring. Next, the dual-gateway architecture and secure communication protocols ensure that network bandwidth or latency does not compromise the real-time nature of the monitoring. Therefore, the collected temperature data are aggregated and displayed on a centralized dashboard, allowing clinicians to monitor all hospitalized patients’ vital signs at a glance, as depicted in Figure 3 [12].

### 2.5. Axillary Thermometer

A commercially available digital axillary thermometer (MT200, Good Life Medical Systems, Inc., Costa Mesa, CA, USA) was the reference standard, consistent with institutional practice for in-hospital temperature monitoring. The device, which measures axillary temperature at eight-hour intervals in the cancer unit, has a measurement range of 32.0–42.0 °C and a reported accuracy of ±0.1 °C within the clinical range of 36.0–39.0 °C. All readings were documented in the hospital’s electronic medical records (EMRs). In patients with hematologic malignancies receiving blood transfusions, who are at elevated risk for transfusion-related infections, temperature monitoring was intensified from 8-hour to 15-min intervals. All reference measurements were performed by trained nurses following a standardized protocol. Timestamped data allowed point-by-point comparison with wearable device outputs, ensuring methodological accuracy.

### 2.6. Evaluation

The clinical effectiveness of the MT100D and RBT system was evaluated across four domains: measurement concordance with the reference, early fever detection rate, fever-triggered clinical response timing, and user satisfaction. Throughout the study, wearable device readouts and alerts were logged but not disclosed to clinical staff, and no treatment decisions were based on the device data. Early-detection metrics were calculated retrospectively to ensure unbiased analysis. Usability assessments were conducted via author-developed paper-based surveys distributed to clinical nurses involved in patient care. Patients also completed questionnaires regarding their experience with the wearable device. All responses were collected after the monitoring period and analyzed using descriptive statistics. Survey instruments and scoring details are described in Supplementary Materials S1 and S2.

There were no indeterminate results in either the index or reference tests, as all temperature readings were continuous numeric values and dichotomized based on the 37.8 °C fever threshold. Missing data were addressed using pre-specified exclusion criteria: patients with fewer than 10 valid measurements or temperatures below 35.5 °C were excluded. No subgroup analyses were planned or conducted; all reported diagnostic accuracy metrics apply to the overall cohort and should be considered exploratory.

### 2.7. Statistical Analysis

The clinical effectiveness of the MT100D and RBT system was evaluated across four domains: measurement concordance with the reference, early fever detection rate, fever-triggered clinical response timing, and user satisfaction. Usability assessments were conducted via author-developed paper-based surveys distributed to clinical nurses involved in patient care. Patients also completed questionnaires regarding their experience with the wearable device. All responses were collected after the monitoring period and analyzed using descriptive statistics. Survey instruments and scoring details are described in Supplementary Materials S1 and S2.

### 2.8. Agreement Evaluation

The discrepancy in body temperature readings was calculated to assess the agreement between the MT100D and the axillary thermometer. Differences between the two measurements are presented as mean ± standard deviation (SD). The agreement was analyzed using intraclass correlation coefficients (ICC) and Bland–Altman analysis (absolute accuracy). A two-way random-effects model calculated ICC values with 95% confidence intervals (CI).

A Bland–Altman plot with 95% limits of agreement (LoAs) was generated to visualize the measurement agreement. This method compares two measurement techniques [20] by plotting the differences against one of the methods, typically the reference standard. The plot includes three lines: the mean difference and the upper and lower LoAs, calculated as mean difference ±1.96 SD. If most data points fall within the 95% CI limits of the LoA, the two devices are considered to agree with 95% CI.

### 2.9. Fever Detection

The MT100D’s fever detection performance was assessed against the reference axillary thermometer. Fever detection was performed in two stages: (i) static fever detection and (ii) early fever detection. Static detection refers to identifying fever at a specific time point based on an immediate temperature reading. The performance was evaluated using sensitivity, specificity, and the area under the receiver operating characteristic curve (AUROC), based on a fever threshold of ≥37.8 °C [21], consistent with clinical guidelines for FN in those immunocompromised. This threshold was assigned to true positive, false positive, true negative and false negative classifications.

Early fever detection was assessed by measuring the time difference between fever onset detected by the MT100D and its subsequent identification by the axillary thermometer. This interval was used to determine the MT100D’s capacity for early fever detection. For static diagnostic performance, the clinical fever threshold was set at 37.8 °C. To derive the AUROC, the wearable temperature threshold was varied across a continuous range (36.0–39.5 °C) using synchronized MT100D–reference data, and corresponding sensitivity and specificity values were plotted to generate the ROC curve.

### 2.10. Assessment of Satisfaction and Usability

User satisfaction and system usability were evaluated using a structured questionnaire rated on a five-point Likert scale, as shown in Supplementary Materials Files S1 and S2. The survey assessed subjective perceptions of convenience, ease of use, and discomfort related to the MT100D and the RBT monitoring system.

Patients completed a seven-item questionnaire. Questions 3–5 were negatively phrased and reverse-coded during analysis. For example, if a patient’s response was 1, it was scored 5 for analysis. For clinicians, a more comprehensive 17-item questionnaire was administered to evaluate the MT100D user manual, patch handling, monitoring interface (web system), and overall satisfaction. Detailed survey items and scoring methodologies are provided in Supplementary Materials S1 and S2, respectively.

## 3. Results

### 3.1. Patient Enrollment and Data Inclusion

A total of 50 patients were enrolled in this study. Following the predefined inclusion and exclusion criteria (Figure 3), three patients with fewer than 10 valid clinical records were excluded. Therefore, 47 patients, yielding 4798 temperature records, were included in the final analysis.

### 3.2. Patient Characteristics

A total of 3 of the 50 enrolled patients with 10 or fewer clinical records were excluded, leaving 47 patients for analysis. Participants with the target condition (fever) were diagnosed with various hematologic malignancies, such as acute myeloid leukemia (AML), acute lymphoblastic leukemia (ALL), and myelodysplastic syndrome (MDS). All patients were receiving intensive chemotherapy and other regimens known to cause severe neutropenia. Therefore, regardless of disease subtype or severity, all febrile participants were uniformly classified as high-risk for febrile neutropenia and related complications.

Table 1 summarizes the baseline characteristics of all enrolled participants. The median age was 52 years (6–88 years), and 23 (46%) were male. Among the 50 patients, 39 (78%) had AML, six (12%) had ALL, one (2%) had MDS, one (2%) had severe aplastic anemia, and three (6%) had brain tumors. The median hospitalization duration was 14 days (interquartile range: 6–24 days), ranging from 1 to 47 days. Comorbidity data were partially available but not consistently documented. Since all participants were hospitalized for hematologic or solid tumors and were at uniformly high risk of febrile neutropenia due to their treatment, the presence of chronic comorbidities was not considered to substantially affect fever risk or the diagnostic performance of the MT100D. No alternative diagnoses were recorded among the 21 patients who did not develop a febrile episode. All patients remained under close observation for potential febrile events, and the absence of fever was attributed to clinical stability rather than underlying conditions. All patients were neutropenic due to either chemotherapy or underlying disease, placing them at consistent risk for FN. While the study did not systematically document the exact timestamps for clinical interventions (such as antipyretics, antibiotics, or blood cultures), all patients were managed in a hematologic cancer ward with a standardized fever management protocol. Once fever was confirmed—regardless of detection method—interventions were promptly initiated per institutional protocol.

### 3.3. Body Temperature Agreement Performance

**Figure 4:**
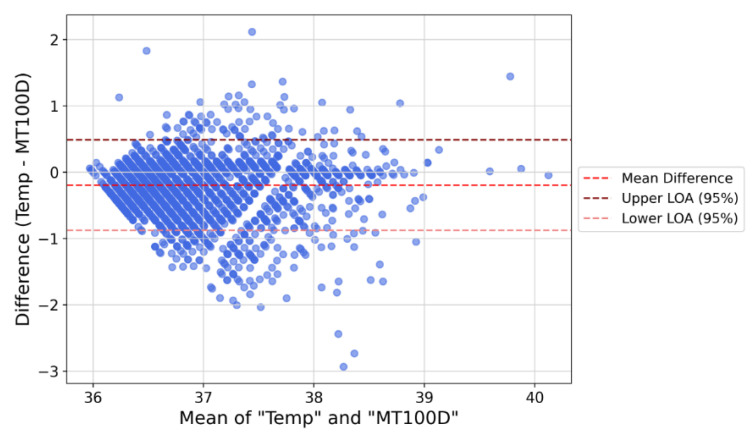
A Bland–Altman plot of all data pairs.

To evaluate the agreement between the MT100D wearable patch and the standard axillary thermometer, the mean temperature difference was calculated as −0.19 ± 0.35 °C. Reliability testing revealed good concordance between devices, with an ICC of 0.78, indicating strong consistency. Figure 4 presents the Bland–Altman plot of all data pairs. The temperature differences did not vary significantly with measurement magnitude, further supporting the agreement. An ICC value above 0.7 generally indicates good agreement [22], confirming MT100D’s performance in replicating standard temperature measurements.

### 3.4. Fever Detection Performance

As shown in Table 2, 24 of the 45 patients included in the analysis (53.3%) were classified as high-risk and experienced febrile episodes. Among these 24 patients with fever, 82.07% (151/184 fever events) required medication, necessitating close clinical monitoring. The MT100D-based wearable RBT monitoring system demonstrated high performance in static fever detection, especially within high-risk populations. The system achieved a sensitivity of 81.5% (95% confidence interval [CI]: 76.8–85.6%), specificity of 96.3% (95% CI: 95.7–96.8%), and AUROC of 96.6% (95% CI: 95.6–97.4%). A cross-tabulation of detection outcomes between the MT100D and axillary thermometer is presented in Supplementary Material S3.

**Figure 5:**
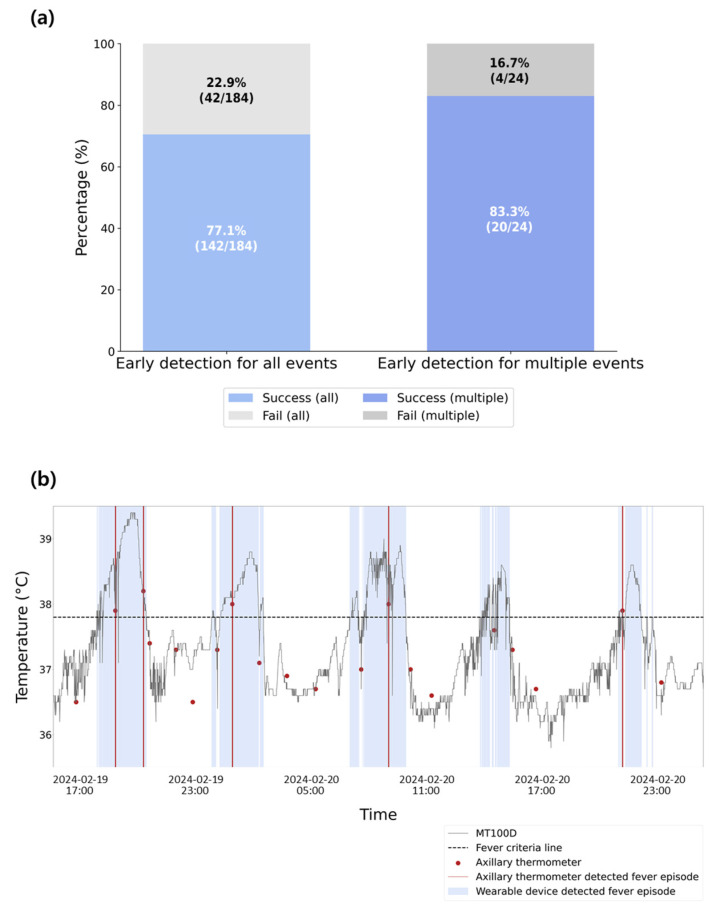
Early fever detection analysis. (**a**) Early fever detection rates for all febrile events (**left**) and multiple febrile episodes (**right**). (**b**) Representative clinical case illustrating early fever detection by the MT100D compared to the reference device.

The success rate and the time difference in fever identification between the MT100D and the reference thermometer were evaluated to assess early detection capability. Figure 5a (left) shows that the early fever detection success rate was 77.1%. Furthermore, the MT100D detected fever on an average of 1.13 ± 1.28 h earlier than the axillary thermometer.

Among the 29 patients who experienced at least one febrile episode, 24 had prolonged hospital stays and were considered as high risk for recurrent fever. These 24 patients contributed to the majority of multiple fever events. The system successfully detected 83.3% (20/24) of these repeated episodes early, as shown in the right panel of Figure 5a. A representative case of early fever detection using the MT100D compared to the reference thermometer is illustrated in Figure 5b.

### 3.5. Adverse Event Analysis

No adverse events were reported with the reference device (axillary thermometer), a noninvasive, routine clinical instrument. For the MT100D wearable patch, five participants (10.6%) reported mild skin irritation, such as itching or redness, and seven participants (14.9%) expressed wanting to remove the patch during use. All events were self-limited and required no medical intervention.

### 3.6. Satisfaction and Usability Results

To assess the usability and satisfaction of the MT100D, patients and healthcare professionals completed structured surveys using a 5-point Likert scale. Of the 47 participants, 45 completed a seven-item questionnaire evaluating ease of use, comfort, and skin-related issues. Table 3 summarizes the average scores per item.

Patients reported high satisfaction, with ease of use scoring an average of 4.74.

Most participants did not experience significant discomfort, although 10.6% (5/47) reported itching or irritation, and 14.9% (7/47) reported a desire to remove the patch during use.

Nineteen clinicians participated in a 17-item survey assessing the usability, anticipated effort, satisfaction, and likelihood of recommending the MT100D and associated RBT monitoring system. Key results are shown in Table 4. Specifically, Question 16, “I am overall satisfied with the MT100D,” yielded a mean score of 3.47. Question 17, “I would recommend the MT100D to others,” scored 3.53. Performance expectation and ease-of-use questions (such as Questions 10, 11, 13, and 15) consistently scored around 3.5 to 3.7, indicating moderate satisfaction and usability.

## 4. Discussion

### 4.1. Clinical Significance

To the best of our knowledge, this clinical investigation is the first to assess the reliability, accuracy, and usability of a wireless MT100D-based RBT monitoring system for fever detection in pediatric and adult patients with hematologic and solid cancers undergoing chemotherapy. Regarding reliability, the MT100D demonstrated satisfactory agreement with the standard axillary temperature measurements of trained nurses, as evidenced by an ICC of 0.78. This finding supports the reliability of the MT100D-based system across a broad patient population.

Regarding accuracy, the system achieved acceptable performance in static fever detection and early fever identification. These results were derived from a high-risk cohort with recurrent febrile episodes, highlighting the device’s potential to alleviate the burden on medical staff who would otherwise need to measure temperature frequently, especially in patients requiring intensive monitoring. All patients were hospitalized in a hematology unit operating under a standardized fever management protocol. Once fever was clinically confirmed, interventions were initiated promptly regardless of the detection method. Although there was no delay in treatment, the MT100D enabled earlier fever recognition, which may support even more timely clinical responses in future applications.

Furthermore, regarding usability, clinicians and patient caregivers reported high satisfaction with the MT100D and the contactless RBT monitoring system. Survey responses indicated satisfaction levels exceeding 80%, significantly higher than those associated with conventional temperature monitoring methods. When asked whether they would use the device again, participants gave an average score of 4 out of 5, suggesting a substantial likelihood of continued use. Therefore, clinicians favored the MT100D, underscoring its practical value in routine patient care.

This study’s MT100D-based RBT monitoring system allows medical personnel to view real-time temperature data for all hospitalized patients via a centralized interface. This setup facilitates the prompt identification of fever and other potentially serious conditions while also allowing clinicians to evaluate the risk of clinical deterioration. Furthermore, continuous temperature monitoring is particularly crucial following high-risk interventions, such as blood transfusions in patients with cancer, where infection is a significant concern. Our system effectively identified febrile episodes even after such procedures, supporting the transition from traditional intermittent measurements to continuous surveillance. Patients receiving intensive chemotherapy had a median hospital stay of 14 days (interquartile range: 6–24 days.

While the sample size limited the generalizability of these observations, the data nonetheless highlight the potential value of sustained RBT monitoring for patients receiving intensive chemotherapy, especially given the system’s demonstrated scalability across diverse clinical populations. Continuous monitoring enables early detection of neutropenia-associated fever and may mitigate complications by facilitating timely intervention. Furthermore, our system addresses a key limitation of traditional monitoring methods: the inability to track temperature trends effectively after treatment. By providing uninterrupted data, the MT100D-based RBT monitoring system offers a novel framework for assessing treatment response across cancer types, supporting personalized patient care.

### 4.2. Strengths

This study presents several important strengths. First, it was conducted in a long-term inpatient setting among a diverse cohort of pediatric and adult cancer patients, including those undergoing high-risk treatments such as transfusions. The system’s reliability, accuracy, and clinical utility were validated within this context. A primary strength of continuous RBT monitoring is its ability to detect fever trends early—often before significant clinical signs emerge—even when minor deviations from standard readings minor occur. Second, our RBT monitoring system was demonstrated to reduce dependence on specialized personnel. Traditional post-transfusion protocols require staff to measure their vitals every 15 min. Conversely, our centralized dashboard allows clinicians to track patient temperatures remotely and in real-time, significantly reducing manual workload. For example, the system achieved over 80% accuracy in detecting recurrent febrile events during hospitalization. Third, the centralized monitoring interface supports prompt clinical decision-making by enabling prompt identification of fever and potential infection, reducing the need for frequent room visits. The high satisfaction ratings (>80%) reported by clinicians further underscore the system’s practicality and readiness for implementation in oncology wards with varying patient needs. Moreover, this study fully complies with the Standards for Reporting of Diagnostic Accuracy Studies (STARD) guidelines, enhancing our findings’ transparency, rigor, and reproducibility. The completed STARD checklist is provided in Supplementary Material S4.

### 4.3. Limitations

This study has several limitations. First, it did not evaluate the direct impact of the MT100D-based RBT monitoring system on patient outcomes and healthcare expenditure. Further research is required to enhance fever detection by integrating additional physiological parameters, such as heart rate and blood pressure, into the RBT system. Although all febrile participants were consistently classified as high-risk for FN and infection-related complications, the underlying disease types were heterogeneous. The absence of a standardized clinical grading system for disease severity limited our ability to stratify participants using a uniform severity index. Future research should prioritize the development and use of such standardized classification systems. The patch was applied to the axillary region in all participants to ensure consistent readings.

However, future studies should explore how patch placement may influence temperature measurements across various anatomical sites. While satisfaction and usability scores were generally favorable—indicating minimal effort required to operate the device—scores related to web-based monitoring usability were relatively lower. This discrepancy highlights an opportunity to improve the user interface to enhance overall satisfaction. Moreover, the system’s dual-gateway configuration is pivotal in mitigating signal loss caused by patient movement, an issue common in single-connection systems. Despite its effectiveness, implementing this dual-gateway approach may be challenging in healthcare settings lacking sufficient infrastructure or technical support. Broader adoption will, therefore, require further research exploring its scalability and integration across diverse hospital environments.

## 5. Conclusions

This study demonstrated that the MT100D WD strongly agrees with conventional axillary thermometers and that the MT100D-based RBT monitoring system offers high early fever detection rates. These findings support the system’s clinical maturity and suggest its potential for reliable, real-time monitoring of neutropenia-related fever in patients with cancer undergoing chemotherapy.

## Figures and Tables

**Table 1:** Patient characteristics.

Characteristics	Patients (*N^a^* = 47)
Age, median (range)	52 (6–88)
Male sex, *n* (%)	23 (46)
Duration of hospitalization, days (range)	14 (6–24)
Number of patients with febrile episodes, *n* (%)	29 (58)
Disease, *n* (%)	
Acute myeloid leukemia	36 (76.6)
Acute lymphoblastic leukemia	6 (12.8)
Myelodysplastic syndrome	1 (2.1)
Severe aplastic anemia	1 (2.1)
Brain tumor	3 (6.4)

*N^a^*: the number of participants in the study.

**Table 2:** Incidence of fever and static detection performance using MT100D.

Category	Performance (%)
Fever incidence rates among patients	
Fever incidence among hospitalized patients	53.33 (24/45)
Incidence of fever requiring medication	82.07 (151/184)
Static fever detection	
Sensitivity	81.5 (95% CI ^b^: 76.8–85.6)
Specificity	96.3 (95% CI: 95.7–96.8)
AUROC ^a^	96.6 (95% CI: 95.6–97.4)

^a^ AUROC: area under the receiver operating characteristic. ^b^ CI: confidence interval.

**Table 3:** Average scores, standard deviations, and 95% confidence intervals (CIs) for patient responses to the MT100D usability and satisfaction questionnaire.

Question	Mean	Standard Deviation	95% CI ^a^
(1) Applying the patch is more convenient than taking a traditional temperature measurement.	4.74	0.65	±0.19
(2) If I ever need to take the body temperature in the future, I would use this patch again.	4.65	0.82	±0.24
(3) I felt a foreign body sensation while applying the patch.	4.63	0.90	±0.26
(4) The area where the patch was applied was itchy and had skin problems.	4.76	0.77	±0.22
(5) I felt like I wanted to take the patch off while I was putting it on.	4.57	1.05	±0.30
(6) It is easy to remove the patch from the skin.	4.65	0.71	±0.20
(7) No skin problems occurred after removing the patch.	4.80	0.75	±0.22

^a^ CI: confidence interval.

**Table 4:** Average scores, standard deviations, and 95% confidence intervals (CIs) for clinician responses.

Question	Average	Standard Deviation	95% CI ^a^
(1) It is easy to confirm the precautions and warnings regarding the use of the product.	3.58	0.77	±0.35
(2) The instructions for the thermometer are easy to understand.	3.74	0.87	±0.39
(3) The product is easy to use overall.	3.74	0.87	±0.39
(4) It is easy to combine the core module with the product.	3.84	1.01	±0.46
(5) It is easy to remove the product from the skin.	4.11	0.94	±0.42
(6) It is easy to separate the product from the core module.	4.00	0.94	±0.42
(7) I frequently checked the monitoring webpage.	3.74	0.93	±0.42
(8) It is easy to check measurement values on the monitoring webpage.	2.89	1.05	±0.47
(9) It is easy to check the fever alarm on the monitoring webpage.	3.16	0.90	±0.40
(10) I intend to continue using the MT100D for body temperature measurement even after the study is over.	3.68	0.89	±0.40
(11) I think MT100D is useful for measuring body temperature.	3.53	1.02	±0.46
(12) I expect that the MT100D will allow me to obtain body temperature information quickly.	3.68	0.95	±0.43
(13) Learning how to use the MT100D is easy.	3.68	0.82	±0.37
(14) I can use the MT100D skillfully.	3.63	1.12	±0.50
(15) Using the MT100D is easy and simple.	3.68	1.11	±0.50
(16) I am satisfied with the MT100D overall.	3.47	1.07	±0.48
(17) I would recommend the MT100D to people around me.	3.53	1.02	±0.46

^a^ CI: confidence interval.

## Data Availability

The data presented in this study are available on request from the corresponding author.

## Supplementary Materials

The following supporting information can be downloaded at https://www.mdpi.com/article/10.3390/s25237111/s1, Supplementary Material S1: Usability evaluation for patients; Supplementary Material S2: Usability evaluation for clinicians; Supplementary Material S3: Cross-tabulation of fever detection outcomes using MT100D and axillary thermometer; Supplementary Material S4: STARD Checklist.

## Abbreviations

The following abbreviations are used in this manuscript:

ALL	Acute lymphoblastic leukemia
AML	Acute myeloid leukemia
ASCT	Autologous stem cell transplantation
AUROC	Area under the receiver operating characteristic curve
CAR-T	Chimeric antigen receptor T-cell
CI	Confidence interval
CTCAE	Common terminology criteria for adverse events
EMR	Electronic medical record
FN	Febrile neutropenia
HCT	Hematopoietic stem cell transplant
ICC	Intraclass correlation coefficient
LoA	Limits of agreement
MDS	Myelodysplastic syndrome
RBT	Real-time body temperature
SD	Standard deviation
STARD	Standards for reporting of diagnostic accuracy studies
WD	Wearable device
