# Supplementary material for: Novel Wearable-Based Real-Time Temperature Monitoring in Hospitals for Febrile Adverse Events in Patients with Cancer: A Prospective Feasibility Study
Source: Sensors (Basel). 2025 Nov 21;25(23):7111. doi: 10.3390/s25237111 (PMC12694504; doi:10.3390/s25237111)
Supplement: Supplementary file 1 [file sensors-25-07111-s001.zip › Supplementary Material S1.pdf]

Supplementary Material S1. Usability evaluation for patients

Usability evaluation of temperature patch

	Strongly disagree	Slightly disagree	Neutral	Slightly agree	Strongly agree
(1) Applying the patch is more convenient than taking a traditional temperature measurement.	1	2	3	4	5
(2) If I ever need to take the body temperature in the future, I would use this patch again.	1	2	3	4	5
(3) I felt a foreign body sensation while applying the patch.	1	2	3	4	5
(4) The area where the patch was applied was itchy and had skin problems.	1	2	3	4	5
(5) I felt like I wanted to take the patch off while I was putting it on.	1	2	3	4	5
(6) It is easy to remove the patch from the skin.	1	2	3	4	5
(7) No skin problems occurred after removing the patch.	1	2	3	4	5
