# Supplementary material for: Novel Wearable-Based Real-Time Temperature Monitoring in Hospitals for Febrile Adverse Events in Patients with Cancer: A Prospective Feasibility Study
Source: Sensors (Basel). 2025 Nov 21;25(23):7111. doi: 10.3390/s25237111 (PMC12694504; doi:10.3390/s25237111)
Supplement: Supplementary file 1 [file sensors-25-07111-s001.zip › Supplementary Material S2.pdf]

Supplementary Material S2. Usability evaluation for clinicians

A. Evaluation of instruction

	Strongly disagree	Slightly disagree	Neutral	Slightly agree	Strongly agree
(1) It is easy to confirm the precautions and warnings regarding the use of the product.	1	2	3	4	5
(2) The instructions for thermometer are easy to understand.	1	2	3	4	5
(3) The product is easy to use overall.	1	2	3	4	5

B. Usability evaluation of temperature patch

	Strongly disagree	Slightly disagree	Neutral	Slightly agree	Strongly agree
(4) It is easy to combine the core module with the product.	1	2	3	4	5
(5) It is easy to remove the product from the skin.	1	2	3	4	5
(6) It is easy to separate the product from the core module.	1	2	3	4	5

C. Usability evaluation of monitoring webpage

	Strongly disagree	Slightly disagree	Neutral	Slightly agree	Strongly agree
(7) I frequently checked the monitoring webpage.	1	2	3	4	5
(8) It is easy to check measurement values on the monitoring webpage.	1	2	3	4	5
(9) It is easy to check the fever alarm on the monitoring webpage.	1	2	3	4	5

D.1. Overall evaluation - Usage intention

	Strongly disagree	Slightly disagree	Neutral	Slightly agree	Strongly agree
(10) I intend to continue using the MT100D for body temperature measurement even after the study is over.	1	2	3	4	5

D.2. Overall evaluation - Performance expectation

	Strongly disagree	Slightly disagree	Neutral	Slightly agree	Strongly agree
(11) I think MT100D is useful for measuring body temperature.	1	2	3	4	5
(12) I expect that the MT100D will allow me to obtain body temperature information quickly.					

D.3. Overall evaluation - Expected effort

	Strongly disagree	Slightly disagree	Neutral	Slightly agree	Strongly agree
(13) Learning how to use the MT100D is easy.	1	2	3	4	5
(14) I can use the MT100D skillfully.					
(15) Using the MT100D is easy and simple.					

D.4. Overall evaluation - Satisfaction

	Strongly disagree	Slightly disagree	Neutral	Slightly agree	Strongly agree
(16) I am satisfied with the MT100D overall.	1	2	3	4	5
(17) l would recommend the MT100D to people around me.					
