# Supplementary material for: Novel Wearable-Based Real-Time Temperature Monitoring in Hospitals for Febrile Adverse Events in Patients with Cancer: A Prospective Feasibility Study
Source: Sensors (Basel). 2025 Nov 21;25(23):7111. doi: 10.3390/s25237111 (PMC12694504; doi:10.3390/s25237111)
Supplement: Supplementary file 1 [file sensors-25-07111-s001.zip › Supplementary Material S3.pdf]

Supplementary Material S3. Cross-tabulation of fever detection outcomes using MT100D and axillary thermometer

To present the distribution of fever detection outcomes, a cross-tabulation between MT100D and reference thermometer was constructed. This allowed derivation of sensitivity, specificity, and AUROC based on true positive, false positive, false negative, and true negative.

	**Axillary Fever**	**Axillary No Fever**
**MT100D Fever**	260 (True positive)	165 (False positive)
**MT100D No Fever**	59 (False negative)	4314 (True negative)
